# Advances of Mechanisms-Related Metabolomics in Parkinson’s Disease

**DOI:** 10.3389/fnins.2021.614251

**Published:** 2021-02-03

**Authors:** Yanyan Zhang, Jie Li, Xiao Zhang, Dongdong Song, Tian Tian

**Affiliations:** Department of Neurology, The First Affiliated Hospital of Zhengzhou University, Zhengzhou, China

**Keywords:** Parkinson disease, metabolomics, genetic mutations, mitochondrial dysfunction, dysbacteriosis

## Abstract

Parkinson’s disease (PD) is a multifactorial disorder characterized by progressively debilitating dopaminergic neurodegeneration in the substantia nigra and the striatum, along with various metabolic dysfunctions and molecular abnormalities. Metabolomics is an emerging study and has been demonstrated to play important roles in describing complex human diseases by integrating endogenous and exogenous sources of alterations. Recently, an increasing amount of research has shown that metabolomics profiling holds great promise in providing unique insights into molecular pathogenesis and could be helpful in identifying candidate biomarkers for clinical detection and therapies of PD. In this review, we briefly summarize recent findings and analyze the application of molecular metabolomics in familial and sporadic PD from genetic mutations, mitochondrial dysfunction, and dysbacteriosis. We also review metabolic biomarkers to assess the functional stage and improve therapeutic strategies to postpone or hinder the disease progression.

## Introduction

As the second most common chronic neurodegenerative disorder after Alzheimer’s disease, Parkinson’s disease (PD) is a multisystemic disease with multiple mechanisms and neurochemical features, affecting around >2% of all persons above 65 years of age and >4% of all persons over the age of 80 (GBD 2015 Neurological Disorders Collaborator Group, 2017; Santos Garcia et al., 2019; Xu et al., 2019). E Ray Dorsey et al. make an important point about the global burden of PD, with the number of affected individuals having risen from 2.5 million in 1990 to 6.1 million in 2016, with projections that by 2050 the number of PD patients will be at 12 million (GBD 2016 Parkinson’s Disease Collaborators, 2018). From an etiological perspective, the two hallmarks and indicators of a definite diagnosis of PD are the deterioration of dopaminergic neurons and the accumulation of intracytoplasmic protein α-Synuclein (α-Syn), called Lewy bodies, in the substantia nigra region of the brain (Spillantini et al., 1997; Spillantini and Goedert, 2018). They are mainly relevant to various neuropathological insults, such as genetic mutants (Kim and Alcalay, 2017), oxidative stress (Puspita et al., 2017), apoptosis, neuroinflammation (De Virgilio et al., 2016; Rocha et al., 2018), mitochondrial dysfunction (Bose and Beal, 2016), disrupting intercellular communication (Hou et al., 2019), endocrine disorders (De Pablo-Fernández et al., 2017), and inhibition of aberrant protein degradation pathways (Tofaris et al., 2001; Spencer et al., 2014; Sugeno et al., 2014). The α-Syn is linked to PD pathology, which possesses prion-like behavior and presents in various throughout the nervous systems before neuronal death and classical symptoms (Grassi et al., 2018; Ma et al., 2019). However, as a multifactorial disease, PD is also influenced by dietary factors (Tufi et al., 2014; Fitzgerald et al., 2017; Lehmann et al., 2017; Zhao et al., 2019), microorganisms (Keshavarzian et al., 2015; Scheperjans et al., 2015), and different environmental elements, such as metal (Kim et al., 2018), neurotoxins (Bove and Perier, 2012), light exposure (Willis et al., 2018), and infection. The disease has complex etiopathogenesis that has still not been fully elucidated. Though four categories of biomarkers have been recommended to confer accurate diagnosis and assess the condition of patients, including clinical symptoms, genetic mutation, pathological, and neuroimaging changes (Delenclos et al., 2016), markers for an early diagnosis and effective treatments of PD are still lacking. Clinically, there is a high rate of misdiagnosis of the disease and clinical accuracy of PD diagnosis is only 76–84%. Therefore, a better understanding of the etiology and pathogenesis, as well as molecular events associated with clinical symptoms, will be significant for early diagnosis and therapeutic strategies.

Metabolomics is an emerging and effective approach used in the identification and discovery of metabolic biomarkers; it relies on the assessment of various biological samples and provides a series of metabolic signatures involving molecular processes that elucidate pathological changes of diseases. The technology links various metabolic molecular mechanisms to neuronal activity alterations, protein changes or genetic mutations, mitochondrial dysfunction, or dysbacteriosis. As an advanced technique of omics, metabolomics can integrate endogenous and exogenous cellular metabolic activities, holding great promise in its ability to probe biochemical details about the pathological status, progression, and treatment of many chronic metabolic diseases, such as cancer, neurodegenerative disease, and kidney disease (Kalim and Rhee, 2017; Willis et al., 2018). Interestingly, an increasing number of scholars devoted to PD research have indicated that metabolomics can be considered as a powerful tool to define biochemical information, detect metabolomic status, and speculate on underlying mechanisms in the disease (Koeth et al., 2013; Pannkuk et al., 2015). Metabolomics’ high-sensitivity and high-throughput properties might support detailed information of the end-product abnormalities arising from interactions between genes, chemicals, protein structure, and various environmental factors. In this respect, metabolomics could be more applicable than other “omics” techniques, including genomics, pharmacogenomics, and transcriptomics, in the qualitative and quantitative analysis of metabolites from cell or biologic specimens to effectively reflect subtle changes of metabolites (Stoessel et al., 2018). Therefore, the introduction of metabolomics in PD research would provide a new solution for seeking underlying metabolic biomarkers for the predication and treatment of the disease.

Considering clinical and experimental findings in pathological mechanisms, we know that multiple mechanisms may contribute to PD pathogenesis. Specially, most studies about the metabolomics of PD mainly focus on gene alterations, energy homeostasis, and redox reactions resulting from mitochondrial dysfunction. Meanwhile, declined antioxidation systems and mitochondrial disorders are also important causes of neuron inflammation and senescence associated with neuropathology (Boland et al., 2018). Updated preclinical evidence indicated that the bidirectional communication between the gut community patterns and the nervous system of the brain, hereto dysbacteriosis, was identified and plays an important role in both the metabolism and pathology of patients with PD. So, in-depth research on the metabolomics regarding potential metabolic indicators and pathways of PD should focus on its effects on pathogenesis and the pathological process.

In this review article, we provide a concise overview on technical methods and related operative procedures in the field of metabolomics. We review recent research on the relationship between metabolomics and neuropathological changes of PD in terms of genetic mutation, mitochondrial dysfunctions, and dysbacteriosis, and also summarize the molecular mechanisms and metabolites underlying pathological signs as promising biomarkers of pathogenesis in both sporadic and familial PD.

## Metabolomics

**FIGURE 1:**
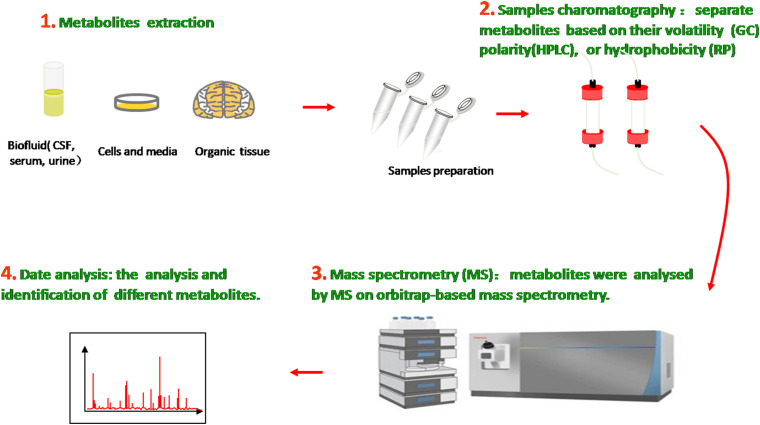
Metabolomics analysis methods. Molecular metabolites were extracted from different samples, ranging from ionic compounds in cell lysates to various organic compositions in plasma, cerebrospinal fluid (CSF), urine, and tissue. Samples chromatography and mass spectrometry were prepared and administrated for analysis and identification.

The metabolome is the entire collection of a wide range of small molecules that participate in body metabolic responses, such as saccharides, amino acids, nucleotides, lipids, and acylcarnitines. Metabolomics, as an analytical technique to investigate disorders in the metabolome of an organism, possess substantial sensitivity, selectivity, and identification capabilities of analyzing diverse varieties of molecular species in biofluids, ranging from ionic compounds in cell lysates to various organic compounds/composition in plasma, cerebrospinal fluid (CSF), urine, and tissue (Figure 1). Compared with traditional targeted approaches, the new untargeted metabolomics have great potential to identify some novel biomarkers and help in indicating the metabolite levels of body fluids, seeking different disease biomarkers to provide useful information about metabolic pathways, metabolites, and pathological mechanisms.

In the past decade, many analytical technologies have been introduced and applied in various metabolomic research fields and thus have furthered the understanding of neurodegenerative diseases on the basis of relevant metabolites as biomarkers. In general, the methodologies used for metabolic identification mainly include proton nuclear magnetic resonance (NMR), magnetic resonance spectroscopy (MRS), liquid chromatography mass spectrometry (LCMS), gas chromatography mass spectrometry (GCMS), flourier transform infrared spectrum (FTIR), and high-performance liquid chromatography (HPLC). The first two metrics utilize the magnetic properties of molecular atomic nuclei in metabolic samples to obtain detailed chemical, structural, and quantities information of metabolites in little samples. By contrast, chromatography mass spectrometry (CMS), which combines the efficient separation capability of chromatography with the high detectability of mass spectrometry, has high analytical precision and superior reproducibility and versatility (Kiraly et al., 2016).

With the development of technologies and current research, there are different groups of metabolic biomarkers used in susceptivity, diagnosis, pharmacodynamic response, and prognostic assessment of diseases (Correia et al., 2017). Emerging evidence has revealed that related metabolomics would be a potential tool for screening and monitoring molecular mechanisms and chemical phenotypes and seeking metabolic signatures as diagnostic and prognostic biomarkers of familial and idiopathic PD. For instance, Bogdanov et al. reported the differences of metabolomic profiling of plasma from idiopathic PD and LRRK2 patients with the G2019S mutation, implicating that the familial PD has unique metabolomic profiles associated with the purine pathway and oxidative processes (Bogdanov et al., 2008; Johansen et al., 2009; Bolner et al., 2011). Similarly, metabolic profiles of blood in idiopathic PD are also different from healthy groups, such as alpha-synuclein, tau protein, urate, and a series of amino acid metabolism (Bolner et al., 2011; Luan et al., 2015; Saiki et al., 2017; Chang et al., 2018; Goldman et al., 2018). These disturbances in the metabolic pathways are related to mitochondrial dysfunctions and the concomitant changes in energy homeostasis and redox reaction, which are thought to be the final common pathways of most endogenous and exogenous factors that are involved in the etiology of PD (Bhinderwala et al., 2019). Recent studies have revealed that there are metabolic differences between treated and drug-naïve PD patients (Bogdanov et al., 2008; Troisi et al., 2019), as well as patients with and without dementia or depression (Hatano et al., 2016; Dong et al., 2018). In addition, biofluids metabolome has potential to distinguish the phenotype of PD. For example, James Roede et al. (2013), used mass spectrometry-based metabolic profiling and showed that polyamine dopamine metabolism was significantly altered in the rapid motor progression of PD compared to both healthy subjects and slow progression PD subjects, which potentially effects of neurodegeneration on neuroinflammation or dopamine metabolism. The metabolomics of animal models demonstrated disturbed metabolic pathways in acylcarnitines, glycerophospholipid, and 4-hydroxypoline in serum, indicating the metabolism influence on the onset and progression of α-Syn pathology (Graham et al., 2018).

## Genetic Metabolomics in PD Patients

**FIGURE 2:**
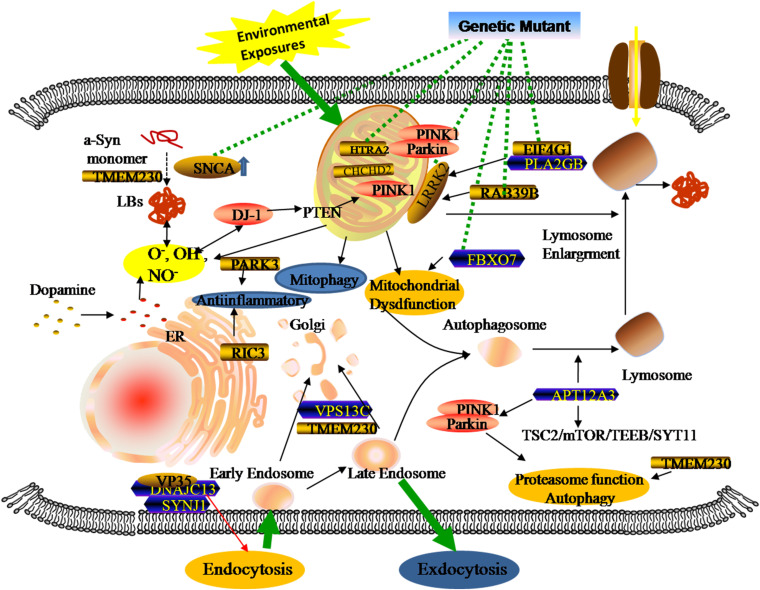
Genetic metabolomics in PD. The complexity of the pathology of PD stemming from the overlap of multiple gene mutants and complex environmental factors. Both locally and systemically, these hazards contribute to a series of responses associated with mitochondrial dysfunction, neuroinflammation, and the failure of clearance mechanisms.

Genomics is the upstream regulator of metabolomics and participates in the modulation of differential metabolite concentrations. Since 1977, studies have provided initial insights into molecular genetics and identified the key contributors that give rise to the occurrence and progression of familial PD cases (Polymeropoulos et al., 1997; Kruger et al., 1998; Braak et al., 2003; Zarranz et al., 2004). Until now, 27 PD-associated genes regions have been identified, affecting 20% of all PD patients (Klein and Westenberger, 2012; Correia et al., 2017; Arkinson and Walden, 2018). There are six genes contributing to the clinically classical form of PD, including three autosomal dominant (SNCA, LRRK2, and VPS35) and three autosomal recessively (PINK1, PARK2, and DJ-1). Additionally, some singular gene mutations are associated with an increased risk of developing PD, including autosomal dominant (*PARK3, GIGYF2, HTRA2, EIF4G1*, RAB39B, *TMEM230, CHCHD2, RIC3*, and *GBA*) and autosomal recessive (*ATP13A2, PLA2GB, FBXO7*, *DNAJC13*, *SYNJ1*, and *VPS13C*). Previous studies revealed that three types of metabolic defects mainly play important roles in the progression of PD: a-Syn protein aggregation, mitochondrial dysfunction, and related oxidative damage. From the structural and functional perspectives, these cellular dysfunctions are associated with different sites and types of alterations in these genes (Figure 2). Even though most familial monogenic forms of PD are identified, metabolic research mainly focuses on the minority of PD related-genes mutations, including *SNCA, LRRK2, PARK2*, and *GBA*. Therefore, a thorough understanding of these gene-related metabolomics will provide available biomarkers for diagnosing and tracking familial PD.

### SNCA

The *SNCA* gene is the first gene to be implicated in PD. Of note, the gene encodes a-Syn protein and its pathogenic mutations were linked with the abnormal accumulation of the presynaptic protein. The initial link between the *SNCA* gene and PD was found by Polymeropoulos et al. (1997) when a missense mutation (A53T) of *SNCA* was implicated in patients with autosomal dominant Parkinsonism from a large Italian family. Shortly thereafter, accumulating evidence has shown the mutations of A30Pro (Kruger et al., 1998), E46K (Zarranz et al., 2004), H50Q, G51D, and A53E in the alpha-synuclein gene (Parajuli et al., 2020). All six-point mutants have been involved in a-Syn overexpression, accumulation, and aggregation, conferring the risk of the disease’s onset or causing familial PD (Singleton et al., 2013). In addition, the SNCA duplication or triplication events (*PARK4* variant), as well as the promoter’s variation, were also involved in the formation of toxic oligomers, misfolded α-Syn, and nigrostriatal denervation, which are vital causes of the disease (Gatto et al., 2010; Koros et al., 2018).

Apart from protein-encoding, research has suggested the role of the SNCA gene in fatty acid synthesis, lipid metabolism (Golovko et al., 2007), mitochondrial membrane composition (Barcelo-Coblijn et al., 2007), and inflammatory responses in the brain (Castagnet et al., 2005; Golovko et al., 2009). Consistent with previous reports, a current study supports previous findings of the SNCA involvement in substance metabolism of the brain. It is worth noting that the authors identified a range of metabolic changes related to the gene through untargeted metabolomic profiling of the brain, such as glycogen depletion, impaired activity of succinate dehydrogenase, and the abnormality of taurine and glutamine (Musgrove et al., 2014). The metabolic alterations not only reflect impaired mitochondrial function in energy production, but also indicate the pathologies associated with other metabolic pathways. Similar to deteriorating metabolic abnormalities in the brain, the *SNCA* gene-related mutations could affect peripheral tissue metabolism in PD patients, which are useful in understanding the metabolic status of the brain and providing molecular signatures. Demonstrated in a cross-sectional study by Heather et al., the premotor A53T *SNCA* carriers have decreasing serotonin transporter densities and serotonergic pathologies compared with healthy controls (Wilson et al., 2019). In addition, the serotonergic abnormalities preceded dopaminergic neuron loss and clinical symptoms, suggesting the potential role of the serotonergic neurotransmitter system in screening and monitoring the progression of the disease (Qamhawi et al., 2015; Wilson et al., 2018). In a longitudinal study, the comparison between A53T transgenic mice and controls revealed that the A53T mutation could substantially increase guanosine levels as a positive regulation against neurodegeneration (Chen et al., 2015).

### LRRK2

*LRRK2* (*Leucine-rich repeat kinase 2*) is the most common gene related to PD, with a frequency of 10% in familiar cases (Paisan-Ruiz et al., 2008; Hernandez et al., 2016). Located in a region on chromosome 12, the gene consists of 51 exons which encode a 2,527 amino acid member of the ROCO protein family (Paisan-Ruiz et al., 2008), and relates to mitochondrial functions, cytoskeletal dynamics, and cellular processes (Guaitoli et al., 2016; Bae and Lee, 2019). Based on current research, eight pathogenic substitutions (p.Arg1441Cys/Gly/His, p.Asn1437His, p.Tyr1699Cys, p.Gly2019Ser, p.Ile2020Thr, and p.Ile2012Thr) and two susceptibility variants (p.Arg1628Pro and p.Gly2385Arg) in *LRRK2* have been identified. The G2019S substitution is most frequent *LRRK2*-related mutation. These PD-associated *LRRK2* mutations might increase intracellular ROS production and contribute to oxidative stress and the loss of dopaminergic neurons.

The correlation between the increase of oxidative stress markers and reduced antioxidant capacity and *LRRK* mutation was assessed in the current study (Loeffler et al., 2017). They measured oxidative stress and antioxidant markers in CSF from *LRRK2*-related PD patients, sporadic patients, and control subjects. Two direct indicators of oxidative stress, the 8-hydroxy-2’-deoxyguanosine (8-OHdG) and 8-isoprostane (8-ISO) concentrations, were increased in LRRK2 patients compared with healthy groups, while antioxidant capacity might decrease during the progression of the disease. Similar to the *SNCA* gene, the metabolomic profiles of low molecular weight substances in PD patients with LRRK2 mutations are also different from idiopathic PD and healthy controls (Johansen et al., 2009). In this study, the *LRRK2* mutation patients showed significantly decreased hypoxanthine, Xanthine, and uric acid in plasma, suggesting the reduction of related antioxidant activities. In addition, several studies have provided evidence that blood levels of uric acid appeared to correlate negatively with the risk for developing PD (Annanmaki et al., 2007; Ascherio et al., 2009; Ou et al., 2017). These suggest that metabolites of the purine pathway play a potential role in elucidating pathogenesis and biomarkers of PD. Like uric acid, *LRRK2* mutation was associated with impaired serine metabolism, showing decreased serine racemase expression and increased serine levels (Nickels et al., 2019). *LRRK2* genes also took part in other metabolic responses, such as Akt signaling, glucose metabolism, or immunity, contributing to the identification of metabolism in *LRRK*-PD (Infante et al., 2015; Wile et al., 2017).

### PINK1 and PARK2

In autosomal recessively PD, *PINK1*, and *PARK2* are associated with the neurodegenerative disorder, which encode the E3 ubiquitin ligase Parkin and the mitochondrial serine/threonine kinase PINK1 that play important roles in mitochondrial quality control and turnover (Arkinson and Walden, 2018). Under normal conditions, PINK1 can phosphorylate and recruit Parkin proteins from the cytoplasm to depolarized mitochondria, then meditate the ubiquitination of mitochondrial outer membrane proteins and activate mitophagy to degrade the ubiquitin mitochondrial proteins mitofusin 1 and 2 (Pickrell and Youle, 2015; Matheoud et al., 2016; Barodia et al., 2017). Similar to autosomal dominant genes, *PINK1 and PARK2* mutations induce metabolomic changes in PD patients. Okuzumi et al. (2019) analyzed serum metabolomics from *Parkin* patients and age-matched controls, and revealed higher levels of oxidized lipids and fatty acid metabolites and lower levels of antioxidant markers in PARK2 patients, suggesting the relationship between the serum/plasma metabolomics and gene dysfunction. Additionally, as a way of ensuring mitochondrial quality control, the mutation effects the elimination of dysfunctional mitochondria that was associated with an increase of mitochondrial stress, manifesting a systemic oxidative stress markers for the pathomechanisms of *Parkin*-mutation patients (Ueno et al., 2020).

### GBA

The most common genetic risk factor for PD is the glucocerebrosidase (GBA) gene, which is located on chromosome 1q21 and contains 11 exons that encode the lysosomal enzyme glucocerebrosidase. In normal cells, the metabolism of glucocerebroside attributes to the efficacy of the glucocerebrosidase (GCase). Reports indicated that GCase not only increases the breakdown of glucocerebroside into glucose and ceramide, but also plays a role in α-Syn degradation (Sidransky and Lopez, 2012; Migdalska-Richards and Schapira, 2016). By contrast, previous studies have shown that the variants of p.E365K and p.T408M in the GBA gene are associated with PD (Liu et al., 2016; Mallett et al., 2016). The *GBA* mutations disturb the function of related lysosomal enzymes, which provoke a-Syn accumulation (Sidransky and Lopez, 2012), disrupt autophagy-lysosome and molecular homeostasis (Uemura et al., 2015), and impair the functional mitochondria by inhibiting mitophagy (Zampieri et al., 2017). For understanding metabolic consequences associated with the *GBA* gene alterations, recent research has detected the CSF of patients with glucocerebrosidase dysfunction, and observed impairments in mitochondrial function and the urea cycle that increased the abundance of several metabolites, such as 1,5-anhydro-D-glucitol, asparagine, ornithine, glutamine, and glycine (Greuel et al., 2020).

## Metabolomics Associated With Idiopathic PD Patients

Most patients are diagnosed as sporadic idiopathic PD as opposed to familial patients, in which environmental hazards play an important role in the pathogenesis of neurodegeneration diseases. The pathogenesis of PD involves complex interactions among multifarious pathomechanisms that include oxidative stress, mitochondrial alterations, inflammatory response, and dysbacteriosis. These pathological changes usually accelerate the truncation (Kahle et al., 2001; Auluck et al., 2002; Liu et al., 2005) and multimerization of misfolding proteins through phospholipid binding, membrane compound altering, and change in the function of molecular chaperones (Tuttle et al., 2016; Gerez et al., 2019). The identification of aberrant biochemistry underlying neuronal degeneration could be an important step toward discovering mechanisms and accurate markers for the diagnosis and therapy of PD. Based on previous studies and updated evidence exploring the metabolomics profiling of biofluids in PD patients, most existing knowledge shows the alteration of different molecular species that mainly focus on genes alterations, energy homeostasis, and redox reaction resulting from mitochondrial dysfunction. Hence, we summarize the progress on metabolomics in idiopathic PD cases and focus on the metabolic biomarkers associated with mitochondrial dysfunction and dysbacteriosis.

## Mitochondrial Dysfunction

**FIGURE 3:**
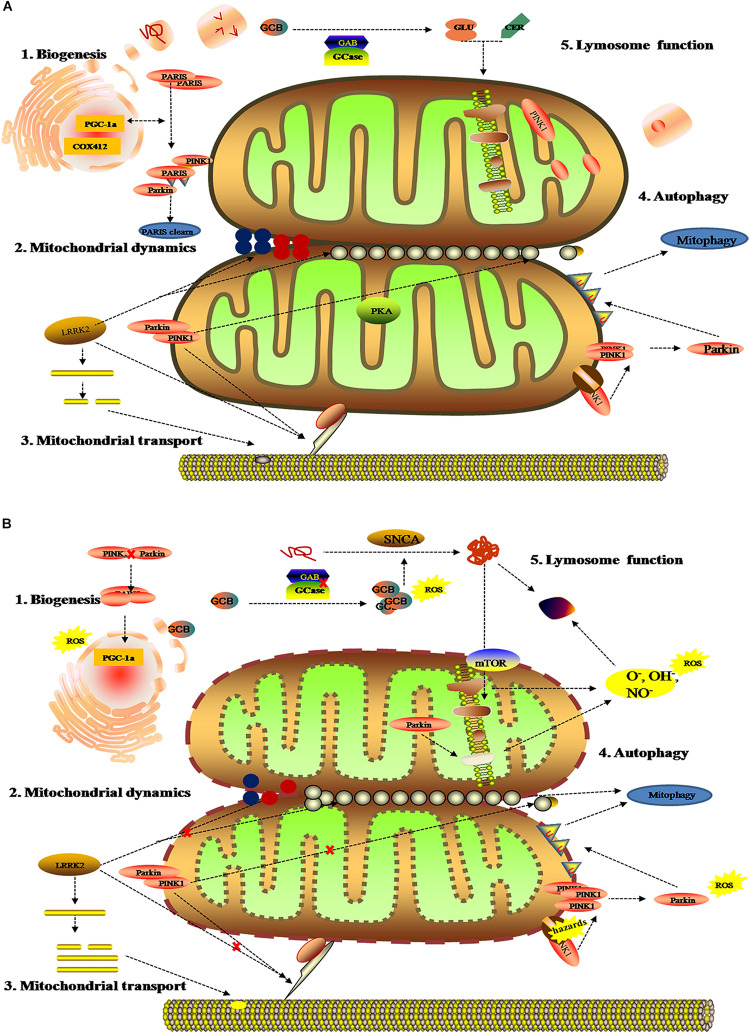
Mitochondrial metabolomics in PD patients. **(A)** Under normal status: 1. mitochondrial complex IV subunit 4 isoform (COX4I2) and proliferator-activated receptor gamma coactivator 1-α (PGC1α) facilitate mitochondrial biogenesis. Additionally, PINK1 and Parkin alleviates PARIS toxicity by phosphorylation and ubiquitination, respectively. 2. PINK1 acts on dynamin-related protein 1 (DRP1) to regulate mitochondrial fission and PKA (PINK1 inhibits protein kinase) inhibits the progress. As such, LRRK2 is also involved in mitochondrial dynamic by MFNs and OPA1 (two mitochondrial fusion proteins) as well as DRP1 (a mitochondrial fission protein). 3. PINK1, Parkin, and LRRK2 mediate mitochondrial transport. 4. PINK1/Parkin clears damaged mitochondria by mitophagy **(B)**. Under gene mutant: 1. mitochondrial biogenesis is inhibited by upregulating PGC1α, which is vulnerable to ROS. 2. The imbalance of mitochondrial dynamics. 3. The mutation of PINK1, Parkin, or LRRK2 halt mitochondrial transportation via Miro, Milton, and motor protein Kinesin-1. In addition, altering LRRK2 expression can stabilize filamentous actin (F-actin) and promote tau neurotoxicity. 4. Hazards causes PINK1 to accumulate when Parkin is impaired, followed by failure in the mitophagy and production of ROS. 5. Deposition of GCB and misfolding a-Syn disrupt mitochondrial respiration, leading to the production of ROS and dysfunction of lysosomes.

As the dynamic powerhouse of a cell, the mitochondrion plays a major role in metabolic activity and generates over 90% of the ATP in a cell. Mitochondria contain their own genomes (mtDNA) and encode vital components associated with mitochondrial function. There is increasing evidence that the mitochondrial function extends well beyond the production of energy in carbohydrate, fatty acid, amino acid, and nucleotide metabolism, it aids in the stabilization of cytosolic calcium, and relates to metabolic pathways, such as the pyruvate oxidation, the Krebs cycle, and various immune responses (Luan et al., 2015; Di Maio et al., 2016). To date, diverse gene mutations and environmental factors have been identified as the cause of mitochondrial dysfunction; it likely to be a key contributor to PD pathogenesis by damaging the transport of mitochondrial proteins, inhibiting respiratory chain function, actuating the generation of reactive oxygen species (ROS), and increasing α-Syn aggregation. As shown in previous studies, the complex I function of the electron transport chain in mitochondrion is impaired because of exposure to environmental toxins such as paraquat, rotenone, and metals (Muthukumaran et al., 2014; Stauch et al., 2016; Thellung et al., 2019). Patients with sporadic PD not only present metabolic changes about abnormal mitochondrial activity in energy homeostasis and redox reaction (Krige et al., 1992; Haas et al., 1995; Penn et al., 1995), but have the presence of mitochondrial oxidative metabolism and insulin resistance (Marcovina et al., 2013; Gonzalez-Casacuberta et al., 2019; Djordjevic et al., 2020). As can be seen in Figure 3, these impaired mitochondrial protein import reduced mitochondrial dynamics, increase ROS, and create mitophagy abnormalities or bioenergetic defects that would deteriorate α-Syn protein misfolding and aggregation.

These mitochondrial changes disturb a series of energy metabolism systems (pentose phosphate pathway, glycolysis, mitochondrial oxidative phosphorylation, glycolysis, acylcarnitines, and the tricarboxylic cycle) (Roede et al., 2013; Trupp et al., 2014; Willkommen et al., 2018), and are also involved in the upregulation or downregulation of amino acids, lipids, and antioxidant substances in PD (Bazinet and Laye, 2014; Lei et al., 2014; Tyurina et al., 2015). Like the correlation between mitochondrial function and gene alterations (Figure 3), comprehensive metabolic analysis of mitochondrial defects arising from environmental factors, such as oxidative stress and energy substance metabolism, might promote the discovery of some discern biomarkers for PD. For example, Younes-Mhenni et al. (2007) and Lewitt et al. (2013) found significantly higher activity of oxidized glutathione, superoxide dismutase (SOD), and catalase in PD patients compared with healthy people. Similarly, increasing 8-hydroxy-2-deoxyguanosine (8-OHdG), an oxidative product of damaged DNA, has also been detected in the blood and urine of PD patients (Roede et al., 2013). On the contrary, the high levels of antioxidants could lower the occurrence and slow the progression of the neurodegenerative disease (Ascherio et al., 2006). Except for metabolic alteration related to mitochondrial oxidation, recent research about metabolomic analysis of cell lysates showed that PD patients present with an increase of lactic acid and a depletion of pyruvic acid and aberrant choline metabolism in extracellular fluid (Amo et al., 2019). Reports have shown that acylcarnitine, as the essential amino acid for fatty acid transport into mitochondria for energy metabolism, was definite in upregulative stages and potentially effected the structure and function of substantia nigra (Mallah et al., 2019).

Notably, some alterations of oxidative stress metabolites might be used to evaluate different subtypes and stages of the disease. Based on CSF and blood samples from patients with PD, Karsten et al. observed specific increases of mannose and fructose, as well as increased threonic acid and reduced dehydroascorbic acid in early-stage PD patients (Trezzi et al., 2017). These changes in oxidation products could reflect the activation of antioxidative stress responses as a resistance mechanism against neuronal injury, in contrast to which, the failure in antioxidant reserve could aggregate the neurodegeneration (Dunn et al., 2014). Significant increases were seen in pyroglutamate and 2-oxoisocaproate and decreases in 3-hydroxyisovalerate, tryptophan, and creatinine, which supported an increase of marks in oxidative responses in preclinical PD (Liu and Wang, 2014). Additionally, some metabolites have also been identified as indicators of the severity of Parkinson’s disease, including uric acid and taurine (Engelborghs et al., 2003).

## Gastrointestinal Dysfunction and Dysbacteriosis

As we all know, gastrointestinal microbes and host usually remain in a mutualistic relationship, in which the microbes keep its diversification and function via the gut to absorb nutrition. In turn, the parasitic microbiota parasitize in the digestive tract and produce a series of biochemical compounds to contribute physical and bioactive barriers or trigger protective immune responses to withstand the effect of exogenous factors (Reza et al., 2019; Parker et al., 2020). Accumulating evidence suggests that many diseases have specific microbiome profiles and potentially communicate mechanisms between the gastrointestinal and the nervous systems, so alterations in gut microbiota have been linked to neurodegeneration, including AD, PD, and Multiple Sclerosis (Sasmita, 2019).

Over the last two decades, neurologists have begun to explore in detail the relationship between the gastrointestinal tract, gut microbiota, and the central nerve systems (CNS). In the last several years, the gut and related microbiome have gained increasing attention because of its close relationship with the etiology of PD. Clinical evidence revealed that neuropathological changes in PD are accompanied by varying symptoms of gastrointestinal dysfunction (indigestion, constipation, bloating, and dysbacteriosis) before the onset of motor symptoms. Experimental evidence showed that bacterial abnormalities and intestinal pathology may play a role in PD symptoms (Fasano et al., 2013; Tan et al., 2015; Van Laar et al., 2019; Mertsalmi et al., 2020). In recent reports, the gut and relevant metabolic products have been given increasing attention because of their importance in the disease pathogenesis (Sampson et al., 2016; Kim et al., 2019), showing that PD patients usually show significant changes in microbiotal abundance and diversity, as well as distinctive profiles of microbiota composition and intestinal metabolites (Keshavarzian et al., 2015; Vascellari et al., 2020; Wallen et al., 2020; Table 1). Although these microbiota composition alterations are heterogeneous, both microbiota disorders and intestinal damage could act as triggering events that lead to dopaminergic loss and pathological a-Syn (Matheoud et al., 2019). Further, numerous experimental and clinical reports indicated that the a-Syn could gather and spread from the gastrointestine to the deep brain (Braak et al., 2003; Kim et al., 2019; Van Den Berge et al., 2019). Notably, the alteration in microbiota abundance was noted in different subtypes and stages of the disease. A study has demonstrated that the abundancy of some microbial compositions, such as Lactococcus, Faecalibacterium, and Leptotrichia, was increased in early-stages of PD, while Comamonas was common in patients with late-onset symptoms. The abundance of Bacteroidetes and Firmicutes were significantly increased in patients with motor-symptoms (Keshavarzian et al., 2015; Lin et al., 2018). Likewise, decreased Prevotellaceae abundance and increased Enterobacteriaceae may have a positive association with intestinal dysfunction in PD patients. Keeping this point in mind, we know that the dysbacteriosis and microbiota metabolomics have potential relevance to the existence of gastrointestinal a-Syn and pathology. The understanding of microbiota metabolomics is essential for exploring the pathogenesis of PD and seeking specific biomarkers that support a more accurate assessment, earlier diagnosis, and better monitoring of the disease progression.

**TABLE 1:** Summary of gut microbiota and their changes in the fecal samples of PD.

Phylum	Family	Genus	Metabolite	Alteration	References
Firmicutes				Down/Up	GBD 2015 Neurological Disorders Collaborator Group, 2017; Xu et al., 2019
	Clostridiaceae	Clostridium	–	Up	Santos Garcia et al., 2019
	Eubacteriaceae	Acetobacterium	–	Up	GBD 2015 Neurological Disorders Collaborator Group, 2017
	Veillonellaceae	Veillonella	–	Up	Xu et al., 2019
	Lachnospiraceae	Anaerostipes	–	Up	GBD 2016 Parkinson’s Disease Collaborators, 2018
		Dorea	–	Down	
		Blautia	–	Down	Spillantini et al., 1997; Xu et al., 2019
		Roseburia	–	Down	Spillantini et al., 1997; Xu et al., 2019
		Coprococcus	–	Down	
		Fusicatenibacter	–	Down	Spillantini et al., 1997
		Faecalibacterium	–	Down	Spillantini and Goedert, 2018
		Lachnospira	Nicotinic acid Pantothenic acid	Down/Up	Spillantini et al., 1997; Kim and Alcalay, 2017; Xu et al., 2019
		Pseudobutyrivibrio	–	Down	Spillantini et al., 1997
	Lactobacillaceae	Lactobacter	–	Up	Kim and Alcalay, 2017; Puspita et al., 2017
	Streptococcaceae	Streptococcus	Cadaverine	Down/Up	Santos Garcia et al., 2019
	Ruminococcaceae	Anaerotruncus	–	Up	GBD 2015 Neurological Disorders Collaborator Group, 2017
Bacteroidetes				Down/Up	Kim and Alcalay, 2017; Santos Garcia et al., 2019
	Bacteroidaceae	Bacteroides	–	Down/Up	Rocha et al., 2018; Santos Garcia et al., 2019
	Odoribacteriaceae	Odoribacter	–	Down	Spillantini et al., 1997; De Virgilio et al., 2016
	Rikenellaceae		–	Down	
	Prevotellaceae	Prevotella	–	Down/Up	Bose and Beal, 2016; Rocha et al., 2018
	Porphyromonas		–	Up	Spillantini et al., 1997
Proteobacteria				Up	De Virgilio et al., 2016; Santos Garcia et al., 2019
	Alcaligenaceae		–	Down	
	Comamonadaceae		–	Down	
	Desulfovibrionaceae	Desulfovibrio	–	Up	
	Desulfohalobiaceae	Desulfonauticus	–	Up	
	Enterobacteriaceae			Down/Up	De Virgilio et al., 2016; Rocha et al., 2018
		Enterobacter	–	Up	
		Escherichia	–	Up	
		Serratia	Nicotinic acid	Up	
		Oscillospira	–	Down/Up	Spillantini et al., 1997; Santos Garcia et al., 2019
		Corynebacterium	–	Up	Spillantini et al., 1997
	Sutterellaceae	Sutterella	–	Down	
	Comamonadaceae	Comamonas	–	Up	
Actinobacteria				Up	Hou et al., 2019
	Bifidobacteriaceae	Bifidobacterium	Pantothenic acid Pyroglutamic acid	Up	GBD 2015 Neurological Disorders Collaborator Group, 2017; Kim and Alcalay, 2017
	Coriobacteriaceae	Slackia	–	Up	
	Microbacteriaceae		–	Up	GBD 2015 Neurological Disorders Collaborator Group, 2017
	Brevibacteriaceae	Brevibacterium	–	Down	Spillantini and Goedert, 2018
Verrucomicrobia				Up	De Virgilio et al., 2016; Santos Garcia et al., 2019; Xu et al., 2019
	Verrucomicrobiaceae	Akkermansia	–	Up	Kim and Alcalay, 2017; Santos Garcia et al., 2019
		Prosthecobacter	–	Up	
Cyanobacteria				Down	Hou et al., 2019
	Aphanizomenonaceae	Dolichospermum	–	Down	

To our knowledge, gut microbiota contributes to host metabolism in the regulation of organic energy metabolism (e.g., lipids, amino acids, and vitamins), as well as to the differentiation and function of immune cells (Cani, 2018). The specific microbial metabolites are disordered when gut microbes are out of balance in abundance and diversity (Table 2). Based on previous studies, the PD-related dysbacteriosis could induce changes in carbohydrate fermentation, protein, and lipid metabolism which could generate SCFA, p-cresol and phenylacetylglutamine, protocatechuic acid, secondary bile acids, and other metabolites (Wahlstrom et al., 2016; Murota et al., 2018; Cirstea et al., 2020). Specifically, the concentration of short chain fatty acids (SCFA) have largely implicated a significant correlation between gut microbiota and PD, and has been implicated as a driver of the onset and progression of PD (Qiao et al., 2020). The SCFA is a metabolic product that possesses anti-inflammatory and anti-microbial function qualities and protects from intestinal permeability, oxidative stress, and immune injury (Donohoe et al., 2011; Sanchez-Guajardo et al., 2015). Further, the SCFA contain a functional composition—Butyrate—that not only supplies the main source of energy for the gut epithelium, but also strengthens the gastrointestinal barrier function (Volf et al., 2016; Agusti et al., 2018). Therefore, the lower abundance of the microbes that produce SCFA could have negative effects for the intestinal barrier and immune function to induce gastrointestinal symptoms of PD, including constipation, intestinal inflammation, and intestinal barrier leakiness (Segain et al., 2000). From what has been discussed above, the metabolic changes of SCFA caused by gut microbial dysbiosis may be a biomarker for better evaluation of PD conditions.

**TABLE 2:** Summary of microbiota cluster and their features in gut.

Cluster		Features	Alteration	References
Opportunistic pathogens	Porphyromonas Prevotella Corynebacterium	NLRP3 inflammasome LPS	Up	Spillantini et al., 1997
SCFA-producing bacteria	Blautia, Roseburia, Coprococcus Dorea Lachnospira Faecalibacterium Oscillospira Corynebacterium	SCFAs-producing Butyrate-producing Vagal activation	Down	Spillantini and Goedert, 2018; Xu et al., 2019
Probiotic bacteria	Lactobacillus Bifidobacteriaceae	Cellulose metabolism	Up	Kim and Alcalay, 2017
Cohesive bacteria	Clostridium Oscillospira Akkermansia Ruminococcaceae	–	Up	Spillantini and Goedert, 2018

## Future Perspectives

Collectively, these findings may mark a new step on the path toward the metabolomics of PD. Paralleling with the availability of test samples and advances in identification technology, metabolomics has been considerably applied as a tool in PD research (Koeth et al., 2013; Pannkuk et al., 2015). However, due to the heterogeneity of humans in regards to genetic expression, dietary habit, environmental exposure, and physical behaviors, only a few specific biomarkers can currently be recommended in clinical practice. Hence, further works on the correlation between metabolomics and the neurodegenerative disease would be valuable. It is of great clinical significance to discover specific biological markers of PD, so as to early screen high-risk populations and facilitate timely diagnosis and reasonable therapeutics.

## Author Contributions

YZ drafted the manuscript. TT was responsible for the design and conception of the work. JL, XZ, and DS participated in the discussion about article writing and revision. All authors read and approved the final manuscript.

## Conflict of Interest

The authors declare that the research was conducted in the absence of any commercial or financial relationships that could be construed as a potential conflict of interest.
